# A novel phenotype of 13q12.3 microdeletion characterized by epilepsy in an Asian child: a case report

**DOI:** 10.1186/s12920-020-00801-1

**Published:** 2020-10-06

**Authors:** Mina Wang, Bin Li, Zehuan Liao, Yu Jia, Yuanbo Fu

**Affiliations:** 1 The Department of Acupuncture and Moxibustion, Beijing Hospital of Traditional Chinese Medicine, Capital Medical University, Beijing Key Laboratory of Acupuncture Neuromodulation, Beijing, 100010 China; 2 Graduate School, Beijing University of Chinese Medicine, Beijing, 100029 China; 3 School of Biological Sciences, Nanyang Technological University, 60 Nanyang Drive, Singapore, 637551 Singapore; 4 Department of Microbiology, Tumor and Cell Biology (MTC), Karolinska Institutet, Biomedicum, Solnavägen 9, 17177 Stockholm, Sweden; 5 Department of Neurology, Xuanwu Hospital, Capital Medical University, Beijing, China

**Keywords:** Epilepsy, 13q12.3, Microdeletion, Case report

## Abstract

**Background:**

The microdeletion of chromosome 13 has been rarely reported. Here, we report a 14-year old Asian female with a de novo microdeletion on 13q12.3.

**Case presentation:**

The child suffered mainly from two types of epileptic seizures: partial onset seizures and myoclonic seizures, accompanied with intellectual disability, developmental delay and minor dysmorphic features. The electroencephalogram disclosed slow waves in bilateral temporal, together with generalized spike-and-slow waves, multiple-spike-and-slow waves and slow waves in bilateral occipitotemporal regions. The exome sequencing showed no pathogenic genetic variation in the patient’s DNA sample. While the single nucleotide polymorphism (SNP) array analysis revealed a de novo microdeletion spanning 2.324 Mb, within the cytogenetic band 13q12.3.

**Conclusions:**

The epilepsy may be associated with the mutation of *KATNAL1* gene or the deletion unmasking a recessive mutation on the other allele, and our findings could provide a phenotypic expansion.

## Background

Epilepsy refers to a chronic neurologic disorder, and it leads to impairments of cognitive and behavioral function. Although the mechanism of epilepsy is still unclear, it is believed that genetic cause is strongly associated with epilepsy of infancy and childhood. Moreover, the genetic cause of epilepsy was initially demonstrated in 2001, with a finding that all seven children in a study of Dravet syndrome had a de novo *SCN1A* mutation. With the development of molecular techniques, more discoveries between genetics and epilepsy have been revealed [1–3]. Besides, chromosome 13 owns one of the lowest gene densities among human chromosomes and structural and functional variations of it may lead to 13q-syndrome, but interstitial deletion of 13q12.3 has only rarely been reported [4].

M. Drummond-Borg et al. [5] have reported a complex chromosome rearrangement involving chromosome 2,13, and 20 in the normal mother of a girl with mild clinical features, developmental delay and an interstitial deletion of 13q12.1-q14.1. Also, another 14 cases of de novo 13q partial deletions (seven terminal and seven interstitial) ranging from 4.2 to 75.7 Mb showed varying degrees of intellectual disability and specific clinical features, among them, 8 had central nervous system anomalies, 6 had eyes abnormalities, 9 had facial dysmorphisms and 10 had hand or feet anomalies [6]. Der Kaloustian et al. [7] have revealed a patient with an interstitial deletion of 2.1 Mb at 13q12.11 who had mild developmental delay, craniofacial dysmorphism, a pectus excavatum, narrow shoulders, malformed toes café-au-lait spots. Furthermore, a child with approximate 12 Mb deletion involving chromosome bands 13q12.3-13q14.11 showed immunodeficiency with elevated IgM levels, mild and transient cerebellar ataxia, and developmental delay [8].

However, currently, there is no report illustrating the correction between microdeletion of 13q12.3 and epilepsy. Here, we present an Asian (Chinese) patient with a microdeletion of 2.324 Mb on 13q12.3, who displayed the recurrent unconsciousness with convulsion for 7 years.

## Case presentation

The proband is a 14-year-old Asian female. She is the first child of nonconsanguineous parents who have another healthy child. The family history was unremarkable. She was delivered by forceps-assisted vaginal delivery at gestational age 40 weeks, with 20 h of labor. Her birth weight was 4250 g. She was not able to suckle for several days after she was born. A developmental delay was observed since her first few months of life. The child had the capability of independent walking at 17 months. At the age of 20 months, she was able to speak. The child occurred hyperpyretic convulsion twice at age of four and a half, and five respectively.

At the age of 7 years, she initially appeared absence, unconsciousness, right skew of head and eyes, head back, tumble, limb convulsion, lips cyanosis, rustle in the throat, sustaining for 2 to 4 min, coupled with headache, emesis and impaired consciousness. This situation occurred 3 to 4 times per year. Electroencephalogram (EEG) was obtained at 8 years old, which showed frequent epileptic discharge in right medial temporal lobe at awake, significantly paroxysmal epileptic discharge in bilateral lobe during sleep, and sporadic epileptic discharge in central and superior lobe during sleep. Then the same attack occurred twice at the ages of 9 and 10 respectively. At the age of 11, the frequency of attack increased to four times per year, and even more regular at age of 12. Therefore, a second EEG was performed at her age of 12 and revealed epileptic discharge in occipital lobe, suspecting her of absence seizure. Meanwhile, brain magnetic resonance imaging (MRI) manifested signal enhancement in bilateral hippocampus on FLAIR sequence. Between 13 and 14 years of age inclusive, epileptic seizures took place about every 7 to 10 days, with rapid vibration of lower limbs occurring dozens of times per day. Thus, a third EEG was performed to the child that illustrated slow waves in bilateral temporal regions, significant in left, with generalized spike-and-slow waves, multiple-spike-and-slow waves and slow waves in bilateral occipitotemporal, significant in left.

After admitting, the physical examination revealed specific facial features with ocular hypertelorism, low insertion of the columella, malar flattening, bend of index and ring fingers and transverse lines in both palms. Cognitive performance was unable to be assessed. Routine metabolic assays were performed the day after admission. Serum sodium valproate: 94.53μg/ml (reference value: 50.0–100.0μg/ml), thyrotropin: 8.30uIU/ml (0.34–5.6uIU/ml, mildly high), fibrinogen: 4.07 g/l (2.0–4.0 g/l, mildly high), alanine aminotransferase: 57iu/L (5.0–40.0iu/L, mildly high), indirect bilirubin: 2.99umol/L (3.42–15.1umol/L, mildly low), γ – glutamyltranspeptidase: 57iu/L (7.0–50.0iu/L, mildly high), and blood ammonia: 70μg/dL (0.0–100.0μg/dL). Moreover, her abdominal ultrasound showed no obvious abnormality in her gall bladder, spleen, pancreas and kidney, while low echo was found in left lobe of liver, which indicated non-uniform fatty liver.

The patient was diagnosed with two types of epileptic seizures: the first type of seizure was partial onset seizures characterized by recurrent absence, unconsciousness, convulsion of four extremities, and aconuresis. The partial onset seizures could last 2 to 4 min and occurred about every 7 to 10 days. The second type of seizure was myoclonic seizures, which manifested as rapid myoclonic jerks of bilateral lower limbs. The frequency of this type of seizure was dozens of times per day. She was provided levetiracetam (43.5 mg/kg/day), lamotrigine (3.0 mg/kg/day), and sodium valproate (14.5 mg/kg/day).

## Genetic study

### Methods

Exome sequencing was performed. The whole area of exons and adjacent area of introns (50 bp) were captured by SeqCap EZ MedExome Kit (Roche Nimblegen) from segmented, spliced, amplified, and purified DNA sample obtained from peripheral blood of the patient. Then, the captured DNA was eluted, amplified and purified, later sequenced by Illumina. Moreover, comparison and identification of genetic variation was used Nextgene V2.3.4 software and UCSC hg19 human reference genomic sequence which collected the coverage of targeted region and average sequencing depth at the same time. The average sequencing depth of targeted area of whole exome sequencing was 100.05X, among which the sequencing depth was more than 20X in 95.35% targeted sequence. Besides, Sanger sequencing was applied to verify the genetic variation reported by abovementioned approach.

SNP array was conducted by Infinium Global Screening Array. Hence, chromosome abnormalities such as the heteroploidy, deletion, repetition, and uniparental disomy of chromosome fragment, of autosomes and sex chromosomes were detected.

### Results

The exome sequencing was normal that no pathogenic genetic variation was detected in the patient’s or her mother’s DNA sample (Supplementary Table 1).

**Fig. 1:**
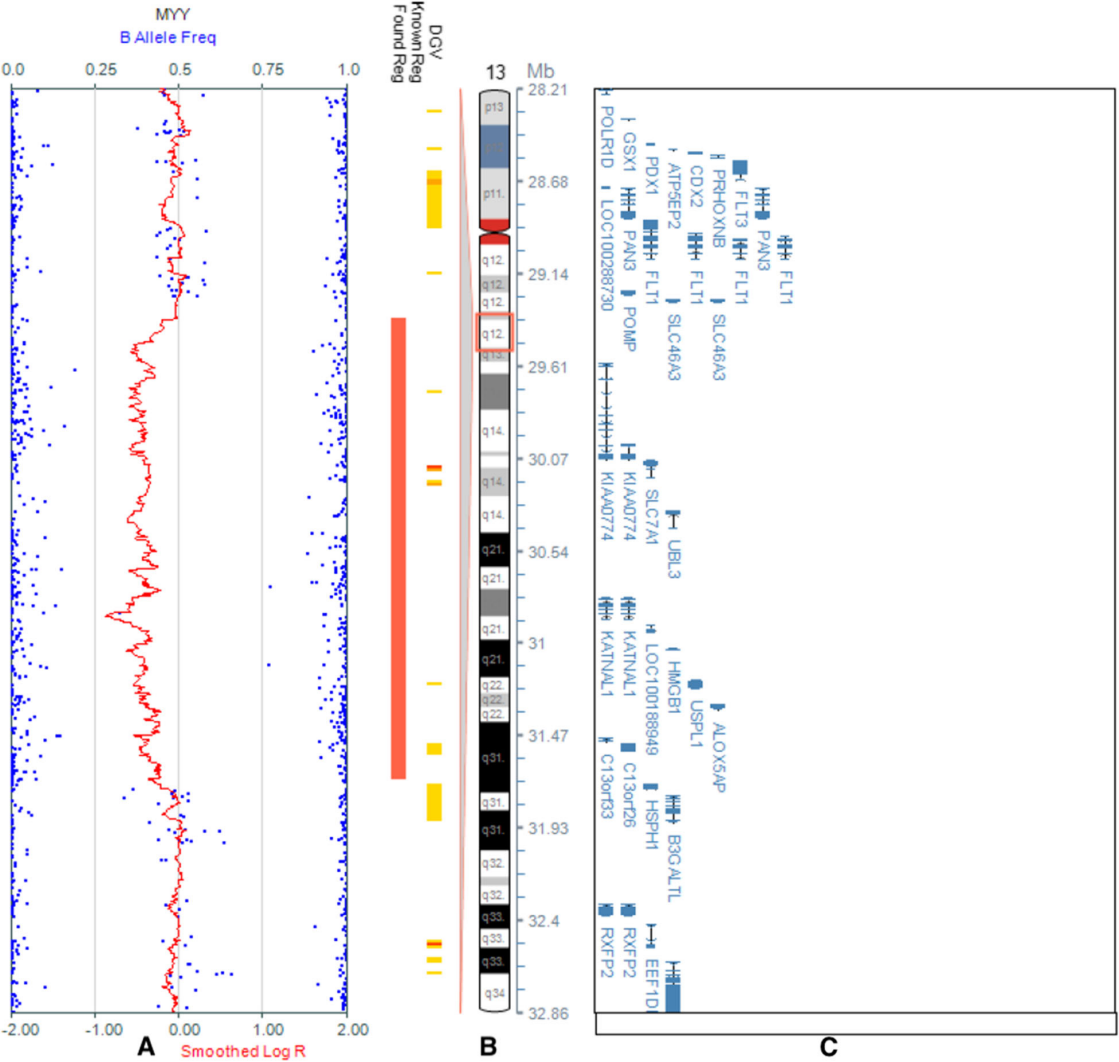
The SNP array of the patient. A: Array-CGH on chromosome 13. B: The ideogram of chromosome 13. C: The genes involved in the 13q12.3 deletion

**Fig. 2:**
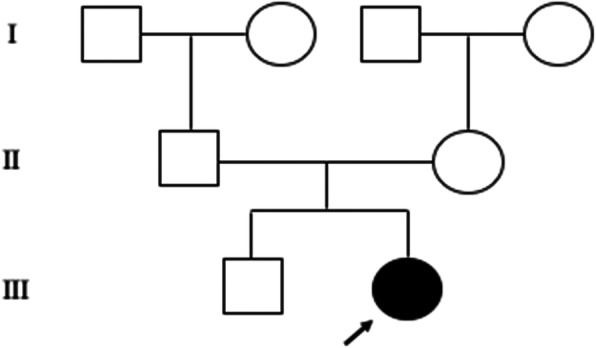
The pedigree of the proband.  normal male,  normal female,  proband

The SNP array (details in Fig. 1) revealed a microdeletion with an approximate size of 2.324 Mb on the 13q12.3 region (arr [hg19] 13q12.3(29,376,209-31,700,395) × 1), which included 9 unique genes: *MTUS2, SLC7A1, UBL3, KATNAL1, HMGB1, USPL1, ALOX5AP, MEDAG3, and TEX26*. However, the mutation was not found in her parents, the pedigree of the proband is presented in Fig. 2.

## Discussion and conclusions

Here, we report a 14-year-old girl being the carrier of a 2.324 Mb microdeletion on 13q12.3 with a novel phenotype: epilepsy. The deletion of chromosome 13q12.3 has been reported only rarely and we merely find three related studies (details shown on Table 1). The clinical phenotypes were complex with facial dysmorphism, hand or feet anomalies, intellectual disability, development delay, and other anomalies. And most of them had poor prognoses. Moreover, an identical mutation site with our case has been found in a study [4] that three probands mainly presented intellectual disability, postnatal microcephaly, and eczema/atopic dermatitis, however, none of them showed epileptic seizures.

**Table 1:** The summary of 3 References of Chromosome 13q12.3 deletions

Reference	The number of cases	Age	Mutation Site	De novo	Clinical phenotype	EEG	Brain CT	Brain MRI
Emilia Cirillo et al. 2012 [8]	One male	17 years	13q12.3–q14.11	Yes	Immunodeficiency with elevated IgM levels, cerebellar ataxia, telangiectasia, freckles, microcephaly, developmental delay, facial dysmorphisms, skeletal anomalies and spontaneous fractures.	Normal.	Moderate enlargement of cisterna magna ventricular system	Moderate hypoplasia of the caudal part of the cerebellar vermis with dilatation of adjacent cerebrospinal fluid spaces.
Deborah Bartholdi et al. 2014 [4]	One male, and two females	Male:18 years Females:9 years and 13 years	13q12.3	Yes	Intellectual disability, postnatal microcephaly, and eczema/atopic dermatitis	N/A	N/A	Normal of two females
Giorgia Mandrile et al. 2014 [23]	One female	5 years	13q12.2q13.1	Yes	Wide set eyes, long philtrum, thin upper lip, and large ears, psychomotor developmental delay and markedly delayed speech	Normal.	N/A	Mild hypomyelination of the subcortical regions and thinning of the corpus callosum.

*N/A* Information not available, *EEG* Electroencephalogram, *CT* Computerized tomography, *MRI* Magnetic resonance imaging

Nevertheless, none of the genes contained in the deleted region are associated with a known monogenic phenotype. *MTUS2* regulates a protein involved in cardiac hypertrophy and neural differentiation, which has been confirmed in mouse and chicken models [9]. *SLC7A1* is related to the function of endothelium and the decrease of nitric oxide (NO) level, it also presents a genetic susceptibility to spontaneous hypertension [10, 11]. *USPL1* represents a third type of SUMO protease, and its expression increases in breast tumor tissue [17, 18]. *ALOX5AP* has been linked to modulate the leukotriene biosynthesis pathway and increase the risk for myocardial infarction, stroke and restenosis [19, 20]. The functions of *UBL3, MEDAG3,* and *TEX26* were not clear. However, currently no evidence has demonstrated the association between these genes and epilepsy (refer to Table 2).

**Table 2:** The summary of 9 Genes Present in the Deleted Region

	Gene	Functions
1	*MTUS2* [9]	Adjusts the development and function of the heart and nervous system in vertebrates
2	*SLC7A1* [10, 11]	Alters endothelial function, Decreases L-arginine and nitric oxide (NO) metabolism, A genetic predisposition to essential hypertension
3	*UBL3*[22]	Not clear, but *UBL3* modification influences protein sorting to small extracellular vesicles
4	*KATNAL1* [12, 13]	Regulates the development of neuronal function and behavior
5	*HMGB1* [14–16]	Encodes a ubiquitous nonhistone chromosomal protein expressed in brain
6	*USPL1* [17, 18]	Represents a third type of SUMO protease, with essential functions in Cajal body biology
7	*ALOX5AP* [19, 20]	Regulates the leukotriene biosynthesis pathway
8	*MEDAG*	N/A
9	*TEX26*	N/A

*N/A* Information not available

We speculate genes that are highly expressed in central nervous system (CNS) are dosage sensitive, which may lead to the occurrence of epilepsy. *HMGB1* and *KATNAL1* are highly expressed in CNS. *HMGB1* is a possible dosage-sensitive gene encoding a ubiquitous nonhistone chromosomal protein expressed in the brain, which regulates inflammatory responses leading to neuronal excitability and seizures [14]. However, it presents a gain-of-function rather than loss-of-function effect. Both animal and human studies have demonstrated that overexpression of *HMGB1* induces epilepsy via regulating TLR4/NF-κB and p38MAPK signaling pathways [15, 16]. Moreover, *KATNAL1* was initially identified with a circadian deficit, it has a similar domain structure as *KATNA1* and the amino acid sequence is 80% identical. Studies has confirmed that loss-of-function effect of *KATNA1* performs reduction of neuronal migration, axonal elongation and axonal branching [12, 21]. A recent study has found its association with the development of neuronal function and behavior. As well as multiple morphological abnormalities and defects in neuronal migration and morphology were detected in *KATNAL1* mutant mice [13]. Therefore, we assume that the mutation of *KATNAL1* is one of the reasons why the proband presents epilepsy. On the other hand, it is rather usual to have an epilepsy in children with chromosomal abnormalities, a possibility that this manifestation might be in fact due to the deletion unmasking a recessive mutation on the other allele also cannot be excluded.

We describe a novel phenotype of 13q12.3 microdeletion, characterized by spontaneously recurrent epileptic seizures, as absence, unconscientiousness, convulsion, rapid vibration of lower limbs with facial and volar dysmorphism, intellectual disability, and developmental delay. Our findings could be a phenotypic expansion (a set of observed phenotypic features extended beyond those previously reported in association with a particular locus) of 13q12.3 microdeletion. However, the underlying mechanism between 13q12.3 microdeletion and epilepsy remains unclear, which requires further in vitro and/or in vivo studies.

## Supplementary information

**Additional file 1 Supplementary Table 1**: The results of exome sequencing of the proband and her mother.

**Additional file 2 Supplementary Table 2:** The results of SNP array of the proband.

## Data Availability

The original data analysis reports (in Chinese) provided by the Beijing Zhongtong Lanbo Clinical Research Institute are available at DOI: 10.6084/m9.figshare.12948311 and 10.6084/m9.figshare.12948302. The summarized analysis reports in English (translated from the original reports) can be found in supplementary Table 1 and supplementary Table 2. The hg19 human reference genomic sequence dataset used in our study was from USCS Genome Brower repository, direct web link: http://genome.ucsc.edu/cgi-bin/hgTracks?db=hg19&lastVirtModeType=default&lastVirtModeExtraState=&virtModeType=default&virtMode=0&nonVirtPosition=&position=chrX%3A15578261%2D15621068&hgsid=900320981_ExrDPx8GaC7hku1X7VbmrfpRld4I. Any clarifications regarding this study can be directed to the corresponding author.

## Abbreviations

SNP: Single nucleotide polymorphism
EEG: Electroencephalogram
MRI: Magnetic resonance imaging
CNS: Central nervous system
N/A: Information not available
CT: Computerized tomography
