# Supplementary material for: A novel phenotype of 13q12.3 microdeletion characterized by epilepsy in an Asian child: a case report
Source: BMC Med Genomics. 2020 Oct 6;13:144. doi: 10.1186/s12920-020-00801-1 (PMC7539513; doi:10.1186/s12920-020-00801-1)
Supplement: Supplementary file 1 — Additional file 1 Supplementary Table 1: The results of exome sequencing of the proband and her mother. [file 12920_2020_801_MOESM1_ESM.docx]

**Supplementary Table 1: The results of exome sequencing of the proband and her mother**

| Samples | The peripheral blood of the proband |
| --- | --- |
|  | The peripheral blood of the proband’ mother |
| Methods | Genomic DNA was extracted from the peripheral blood of the subjects. After fragmentation, ligation, amplification and purification, the regions of whole exon and adjacent intron (50bp) of all human genes were captured by SeqCap EZ MedExome Kit (Roche NimbleGen). The captured DNA was sequenced by Illumina after eluting, amplifying and purifying. NextGene V2.3.4 software and UCSC hg19 human reference genomic sequence were used to compare and identify genetic variation of the subjects, meanwhile, the quality parameters such as coverage of targeted region and average sequencing depth were collected. There are more than 20X of sequencing depth in 95.35% targeted sequence, and the average sequencing depth of the targeted region was 100.05x.  In addition, the genetic variation reported by abovementioned method would be confirmed by Sanger sequencing. |
| Results | No pathogenic / suspected pathogenic variation relating to clinical phenotype was detected. |
