# Supplementary material for: A novel phenotype of 13q12.3 microdeletion characterized by epilepsy in an Asian child: a case report
Source: BMC Med Genomics. 2020 Oct 6;13:144. doi: 10.1186/s12920-020-00801-1 (PMC7539513; doi:10.1186/s12920-020-00801-1)
Supplement: Supplementary file 2 — Additional file 2 Supplementary Table 2: The results of SNP array of the proband. [file 12920_2020_801_MOESM2_ESM.docx]

**Supplementary Table 2: The results of SNP array of the proband**

| Samples | The peripheral blood of the proband | | | | | |
| --- | --- | --- | --- | --- | --- | --- |
| Methods | SNP array was conducted by Infinium Global Screening Array to detect chromosome abnormalities such as heteroploidy, deletion, repetition, and uniparental disomy of chromosome fragment in autosomes and sex chromosomes. | | | | | |
| Chip Code | 201622190047_R12C01 | | | | | |
| Results | **Heteroploidy in autosomes** | **Heteroploidy in sex chromosomes**. | **Chimeras** | **Deletion** | **Repetition** | **Loss of Heterozygosity** |
|  | No findings | No findings | No findings | Yes | No findings | No findings |
|  | **Region** | **Onset** | **End** | **Length** | **CNV type** | |
|  | 13q12.3 | 29,376,209 | 31,700,395 | 2,324,186 | 1 | |
|  | **Genes in the deletion region** | *KIAA0774; SLC7A1; UBL3; KATNAL1; LOC100188949; HMGB1; USPL1; ALOX5AP; C13orf33; C13orf26* | | | | |
|  | **Molecular cell karyotype** | arr[hg19] 13q12.3(29,376,209-31,700,395)x1 | | | | |
| Conclusion | The SNP array revealed a microdeletion with an approximate size of 2.324Mb on the 13q12.3 region (arr[hg19] 13q12.3(29,376,209-31,700,395)x1), which included several unique genes: *KIAA0774; SLC7A1; UBL3; KATNAL1; LOC100188949; HMGB1; USPL1; ALOX5AP; C13orf33; C13orf26* | | | | | |
